# Leveraging chemical synthesis to discover metabolites from the gut microbiome

**DOI:** 10.1111/nyas.70004

**Published:** 2025-08-04

**Authors:** Allison J. Keys, Nitesh K. Nandwana, Eyas Alnasser, Emily C. Gentry

**Affiliations:** ^1^ Department of Chemistry, Virginia Tech Blacksburg Virginia USA

**Keywords:** gut microbiome, mass spectrometry, metabolite discovery, metabolomics, organic synthesis, reverse metabolomics, synthetic chemistry

## Abstract

The gut microbiome has the biosynthetic potential to make a variety of secondary metabolites or natural products, which serve as molecular messages between cells and organisms. These chemical signals are capable of affecting physiology and behavior in real time, and are, therefore, bioactive and can exhibit medicinal properties including anticancer, antimicrobial, or immunomodulating activities. It is clearly important to identify signaling molecules in the human gut, but elucidating their chemical structures can be challenging since traditional isolation methods are typically not available. The discovery of microbiome‐related metabolites requires multidisciplinary collaboration, where chemical synthesis often plays an essential role. This review highlights examples where synthetic chemistry was used to study novel metabolites produced by the gut microbiota. In the first part, we describe examples where organic synthesis was utilized in traditional contexts, as a last step for validating structures and sourcing material for biological testing. The final section of this review discusses next‐generation applications for chemical synthesis, where integration with metabolomic or genomic analysis simultaneously uncovers both structural and biological information about small molecules from the gut.

## Graphical abstract

Metabolomics or the study of small molecule metabolism is used to identify compounds produced by the gut microbiota, but chemical annotation is a major bottleneck. Here, we review examples where organic synthesis was leveraged in traditional or next generation workflows to help identify new bioactive gut microbial metabolites.

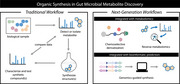

## INTRODUCTION

Small molecule metabolites are central to host–microbe interactions in the gut, but deciphering the language by which host and microbiota communicate is not trivial. Over the past decade, there has been an exciting shift in microbiome‐related research from describing who is there to now answering what are they doing.^1^ Metabolomics has been a key tool in this evolution because it generates snapshots of chemical exchanges over space and time to create a descriptive metabolic picture. For instance, noninvasive, ingestible sampling devices have been used to collect metabolomics and microbiome samples along the human digestive tract, and molecular cartography has been employed to overlay the chemical and microbial composition of diseased lungs onto 3D radiographic images.^2^, ^3^, ^4^ Moreover, time‐resolved metabolomics is often used in clinical studies to observe metabolic differences between disease active and inactive periods or before and after therapeutic intervention.^5^ In this way, metabolomics offers a way toward preventive and personalized medicine, where metabolite levels are collected using noninvasive sampling methods and track health state in real time. However, this plan is ultimately limited by how much chemical space is known and available by synthesis or for purchase. Since the complexity and diversity of the human metabolome is immense, structure annotation is a major bottleneck in identifying secondary metabolites critical for human health.^6^, ^7^, ^8^

Significant challenges arise when trying to chemically identify metabolites from the gut microbiome due to limited material, complex structures, and unstable architectures. When studying the gut microbiota, samples often come from clinical or mouse studies where quantities are not suited for isolation.^9^ Alternatively, cultures of gut bacteria can be used as a source of bioactive microbial metabolites, but rely upon biosynthetic gene expression under laboratory conditions.^10^, ^11^ Genetic manipulation and heterologous expression are generally not well tolerated for scale‐up production in gut anaerobes.^12^ Additionally, some metabolites may elude structure determination because they are reactive or unstable to conditions required for purification.^13^ As such, it is critical to develop isolation‐independent strategies for metabolite discovery that are complementary to traditional workflows.

Mass spectrometry (MS)‐based metabolomics is a well‐suited method for studying the gut microbiome because it provides chemical insights from very small quantities of crude material and does not rely on isolation. However, as mentioned previously, the annotation of metabolites detected is inherently restricted to the chemical space covered by available standards and libraries. For gas chromatography‐mass spectrometry, this is not a problem since it only detects small volatile molecules and the NIST23 library contains electron ionization MS spectra for 346,964 unique compounds, many of which have standardized retention indices for additional validation.^14^ Liquid chromatography‐mass spectrometry (LCMS) experiments, on the other hand, detect thousands of compounds per sample and have smaller, more restrictive libraries available, with NIST23 covering only 51,502 unique compounds in its tandem mass spectral library.

To combat this issue, innovative prediction tools have been created to improve metabolite identification in LCMS data.^15^, ^16^, ^17^ However, even the best workflows provide multiple candidate structures that need to be refined, synthesized, and compared to the detected metabolite before researchers can move forward with more targeted experiments.^18^ Furthermore, training sets of machine learning models for MS/MS spectral identification depend on the known structures available, and prediction capabilities drop off in unfamiliar territory. Ultimately, the biological and ecological roles of gut metabolites cannot be fully examined until their structures are established and chemical materials are available for downstream studies.

Therefore, synthesis bridges a critical gap—linking characterized structures to chemical and biological data. This review highlights examples where synthesis aided the discovery and annotation of bioactive gut microbial metabolites. Here, studies are categorized as either traditional or next‐generation uses of synthesis. Traditional examples provided in this review incorporate synthesis in a conventional way, where it is leveraged to verify structure and access material for bioactivity studies. Conversely, studies classified as next‐generation integrate synthesis into an existing metabolomics or genomics‐based platform for a more comprehensive analysis of metabolic capabilities within an ecosystem or organism. The central topic of this review, organic synthesis, is a well‐established field that has received many accolades for its applications in chemical biology, but the full potential of modern synthetic organic chemistry has not yet been unleashed in the context of the human metabolome. The purpose of this article is to inspire current and prospective synthetic chemists to join forces with analytical chemists and biologists so that together, we may illuminate the unknowns of the human gut.

## TRADITIONAL USES FOR SYNTHESIS

**FIGURE 1:**
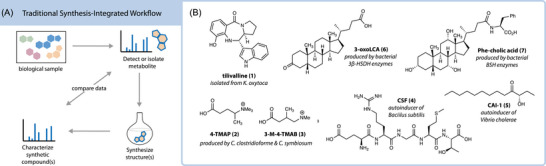
Overview of the traditional use for synthesis in gut microbial metabolite discovery. (A) General workflow where synthesis is used for structure and activity validation. (B) Examples of metabolites discovered by traditional methods that are highlighted in this review.

Traditionally, in natural products research, synthetic chemistry is reserved as a latter step to verify absolute stereochemical structure and supply material for biological assays (Figure 1A). The first section of this review covers cases where chemical synthesis was used in traditional ways to validate structures from the gut microbiota (Figure 1B).

### Tilimycins

Tilimycins are pyrrolobenzodiazepine natural products that were initially isolated and synthesized by Mohr and Budzikiewicz in 1982.^19^ The discovery of tilivalline **(1)** represents the first example where synthetic organic chemistry was used to investigate metabolites produced by the gut microbiota. Secreted by pathogenic bacteria, these enterotoxins are cytotoxic to human cells and can disrupt gut epithelial function.^20^ Tilivalline induces intestinal cell apoptosis in antibiotic‐associated hemorrhagic colitis (AAHC) mouse models through direct DNA damage or microtubule stabilization.^21^ In its initial discovery, tilivalline was purified from cultures of *Klebsiella pneumoniae* var. oxytoca, now known to be a causative agent of AAHC.^22^ Organic synthesis was used to confirm its chemical structure and yielded undesirable 8:92 selectivity favoring the wrong epimer, but authentic standards were accessed nonetheless to compare with native metabolites.^19^ Following this discovery, tilimycins became classic targets for synthetic chemists due to their structural complexities and potent bioactivities. Shioiri and coworkers developed the first stereoselective synthesis of tilivalline in 1986, which showcased a key Mannich‐type cyclization.^23^ Since then, tilimycin production has been further optimized by synthetic and biosynthetic chemists, leading to a better understanding of its bioactivity and ecological function.^24^, ^25^, ^26^

### Carnitine derivatives

Even when structures are not as complex as tilimycins, comparison to authentic standards remains a key component to structure validation of unknown metabolites, especially in cases where there are multiple possible regio‐ and stereo‐isomers. For example, synthesis recently helped identify novel carnitine mimics produced by the gut microbiota. Hulme et al. reported unique chemical analogs of carnitine with mass‐to‐charge ratio (*m/z*) of 160.133, which were detected exclusively in specific pathogen‐free (SPF) mice and absent in germ‐free (GF) mice.^27^ These carnitine derivatives were detected in the gut and brain of SPF mice using matrix‐assisted laser desorption/ionization (MALDI) and desorption electrospray ionization (DESI) mass spectrometry. Cultures of murine gut bacterial isolates were analyzed by MS, finding that two related strains of *Clostridium* produced the same *m/z* 160.133 signal as was seen in the SPF mice. While a correct chemical formula could be obtained from these experiments, further studies were needed to provide absolute structure. Using a combination of nuclear magnetic resonance (NMR), tandem mass spectrometry (MS/MS), and chemical synthesis experiments, the *m/z* 160.133 signal was determined to be 4‐(trimethylammonio)pentanoate (4‐TMAP) **(2)** and 3‐methyl‐4‐(trimethylammonio)butanoate (3‐M‐4‐TMAB) **(3)**. Notably, both of these carnitine derivatives lack the hydroxyl group of native carnitine, which is the functional site for fatty acid conjugation. Therefore, the presence of 3‐M‐4‐TMAB and 4‐TMAP can directly affect fatty acid transport and disrupt normal mitochondrial function. Though this example is interesting not for its use of organic chemistry but rather its biological implications, it is highlighted here to demonstrate how synthesis can be integral to the structure elucidation of gut microbial metabolites. Previous studies had reported a precursor ion with *m/z* 160.133 as a potential biomarker for diabetes, preeclampsia, and nephropathy, but structure annotation was not validated in these reports.^28^, ^29^, ^30^

### Quorum‐sensing metabolites

Quorum‐sensing (QS) molecules, also known as autoinducers, are secondary metabolites produced by bacteria to communicate and coordinate behavior. Autoinduction in microbial communities was first reported in bioluminescence mechanisms for marine bacterial *Vibrio* species.^31^, ^32^, ^33^ More recently, QS metabolites have been investigated in the human gut where they can directly injure epithelial cells or affect the composition of the gut microbial community.^34^, ^35^, ^36^ The structures of QS metabolites found in the human gut range from simple acyl homoserine lactones (AHLs) to more complex, stereodefined peptides. Biosynthesis and genomic techniques are integral to the identification of QS metabolites, but synthetic organic chemistry is commonly employed alongside as a complementary tool. For example, Shaw et al. synthesized a variety of authentic AHLs as standards for a thin‐layer chromatography assay to detect AHL‐producing bacteria.^37^ Synthetic standards were also used to validate the alpha hydroxy ketone structure of autoinducer CAI‐1 **(4)** from *Vibrio cholerae*, the pathogen responsible for cholera, and explore its reactive enamine biosynthetic precursors.^38^ Additionally, a QS pentapeptide competence and sporulation factor (CSF) **(5)** was detected in cultures of *B. subtilis* where its concentration controls competence and sporulation gene expression, ultimately affecting bacterial growth.^39^ Subsequent studies used synthetic CSF to determine that this bacterial peptide not only impacts the gut microbial community, but also induces host intestinal cell expression of heat shock proteins, protecting the intestinal epithelium from oxidative damage.^40^ Taken together, these findings demonstrate how organic synthesis can provide powerful molecular insights into QS mechanisms and consequences in the gut.

### Bile acid metabolites

Bile acids are a class of host‐derived steroidal metabolites released into the small intestine for fat digestion, whose structures are readily modified by gut microbes in an effort to decrease toxicity. For example, some gut microbiota change the stereochemistry or epimerize the 7α‐hydroxy group in chenodeoxycholic acid to decrease its toxicity.^41^ Though the structures of bile acids have been studied for more than 150 years, new structures are still being unearthed with improving detection and annotation technologies. Building evidence suggests that bile acid cometabolism is critically important for human health, so it is essential to identify the structures and downstream effects of microbial bile acid metabolites. Here, we discuss how the various metabolic reactions involving bile acids, namely, oxidation, sulfation, and amidation, were studied using synthesis in a traditional sense to validate structures and provide materials for bioactivity assays.

Oxidation of bile acids is a metabolic transformation that can be performed in host cells as well as gut bacteria possessing the 7α‐hydroxysteroid dehydrogenase gene. This metabolite was recently made using synthesis and then its biological properties were explored in immune cell assays.^42^ Bleach and acetic acid were used to oxidize commercially available lithocholic acid (LCA) to 3‐oxo LCA **(6)**, and this product was also conjugated with glycine. Furthermore, the authors epimerized LCA to isoLCA in this report via activation of the hydroxyl group with a trifluoroacetoxy group followed by an SN2 attack with hydroxide. This example demonstrates how synthesis can be leveraged to manipulate the oxidation state or stereochemistry of metabolites.

Recently, Devlin and coworkers also discovered that gut bacteria can sulfonate steroidal structures. In terms of bile acids, only sulfonation of isolithocholic acid isomers was detected in their study, but they demonstrated *Bacteroidetes* can broadly perform sulfonation on steroids containing a fused A/B ring system.^43^ This includes phytosterol and cholesterol substrates as well as adrenal and sex hormone precursor metabolites. The structures of these microbially derived compounds were verified using synthetic standards created via the reaction of each substrate with excess amounts of a sulfur trioxide‐pyridine complex. Sulfonation is a common Phase II host excretion pathway as it increases water solubility and decreases intestinal absorption of metabolites, but here, it was determined that bacteria can also perform this transformation.^44^

Similarly, the amidation of bile acids can be either a host or microbial transformation depending on the substrate used. Conjugation with glycine and taurine is performed by the host liver, but other amine conjugations can be performed by the gut microbiota. First reported by Quinn et al., the structures of Ile/Leu‐, Phe‐, and Tyr‐microbially conjugated bile acids (MCBAs) **(7)** were discovered through molecular networking and 3D mapping of tandem MS data in SPF versus GF mice.^45^ In this study, synthesis was used to both validate the structures and provide compounds for subsequent biological testing. Since then, bile amidates derived from other amino acids and biogenic amines have been described, and their structures were also confirmed using synthesis.^46^, ^47^

As highlighted here, synthetic chemistry is commonly used as a final step to authenticate structures from the gut microbiota and empower subsequent biological studies. While this is an important and continual role for organic synthesis, it can also play a more integral part in the discovery workflow as described in the next portion of the review.

## NEXT‐GENERATION INTEGRATION OF SYNTHESIS

**FIGURE 2:**
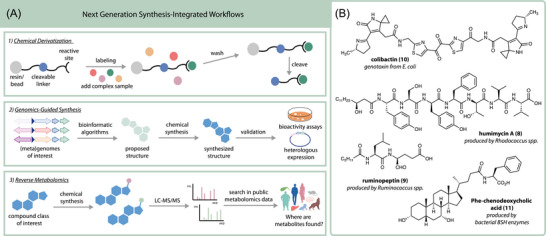
Overview of next‐generation methods integrating synthesis with omics technology. (A) Different strategies currently available across three types of workflows. (B) Chemical structures highlighted in this review that were discovered using one of the three types of next‐generation technologies.

In this section, examples are presented where synthetic organic chemistry is intimately integrated with existing metabolomics or genomics workflows to gain deeper information about metabolites beyond structure. In the previous section, synthesis was not in the spotlight but was rather a supplementary technique, often limited to the supporting information. However, synthesis can play a much larger role, where it is intimately woven into the discovery process. This section highlights innovative next‐generation uses of synthesis, where chemical transformations have been performed in combination with genomics or metabolomics technology to enable structural and biological annotation of gut microbial metabolites. Here, three different approaches are covered: chemoselective derivatization, genomics‐guided synthesis, and reverse metabolomics (Figure 2A). Examples are provided along the way to demonstrate the utility of each method (Figure 2B).

### Chemoselective probes for metabolite enrichment

Synthetic derivatization of molecules prior to analysis has been a strategy implemented in gas chromatography for decades to affect the volatility of analytes and influence fragmentation behavior.^48^ More recently, chemical derivatization was adapted to LCMS‐based workflows to promote ionization, improve limits of detection, and increase chromatographic separation.^49^, ^50^, ^51^, ^52^ Using synthetic chemical probes optimized for unique functionalities, metabolites can be readily tagged, detected, and classified by functional groups.^53^ These derivatizations are performed alongside traditional untargeted metabolomics workflows to assist in compound identification and detection of metabolites that are differentially abundant in control versus test groups.

In modern workflows using cleavable linkers, a chemical probe is developed with three distinct steps in mind—labeling, washing, and releasing—to enrich the analysis mixture and enhance detection (Figure 2A, top). The labeling step is powered by synthetic chemistry, where chemical derivatization is performed on a complex sample using a synthesized cleavable linker system. While structures vary based on utility, each chemical probe contains four general areas: magnetic beads or solid support resin, a cleavage linker, reactive sites, and modification groups. Once desired metabolites have reacted with the chemical probe, a washing process is performed to remove untagged or undesired compounds. A final release step cleaves the metabolite attached to a defined part of the linker system from the resin or bead. The known molecular tag can act as a handle for convenient detection of derivatized metabolites using MS1 or MS2 signals.

While not applied to study the gut microbiome, metabolite enrichment by tagging and proteolytic release (METPR) systems were the first synthetic enrichment platforms designed to improve LC‐MS data detection of low abundance, small biomolecules in complex matrices. Inspired by previous work in activity‐based protein profiling, Carlson and Cravatt developed a complementary set of four resin‐bound chemical probes, each uniquely targeting specific functional groups—amines, carboxylic acids, thiols or aldehydes, and ketones.^51^ This analytical approach was first tested and optimized using known standards, and then its utility was demonstrated in the context of cancer cell metabolomics. Carboxylic acids were the most captured metabolites, with over 250 tagged molecules detected, while ketones and aldehydes were the least detected compound class. Remarkably, their strategy was able to quantify very small, polar metabolites like pyruvate and methyl glyoxal using LC‐MS, which was previously not achievable. The authors determined that the METPR platform provided similar fold changes for cysteine and glutathione levels in cancer cells upon treatment with *N*‐acetyl‐l‐cysteine, when compared to values using traditional metabolomics methods. This approach demonstrated that chemical probes can be used to tag, enrich, and compare relative abundances of a variety of metabolites in complex samples.

Globisch and coworkers later synthesized a *para* nitrocinnamyloxycarbonyl (Noc)‐based cleavable linker for capturing amine‐containing groups in human fecal, urine, and plasma samples.^54^ Using this strategy, they detected ammonia, 2‐amino‐3‐methylbenzoic acid, 1‐aminoacetone, and 1‐aminopropan‐2‐ol in human feces using LCMS for the first time. Notably, authors were able to quantify gaseous molecules like ammonia and methylamine as well as other volatile microbiome‐derived amines using this system. Their group also optimized chemoselective probes for ketone‐, aldehyde‐, and thiol‐containing metabolites in complex samples.^55^, ^56^, ^57^ A boronate ester‐based linker was later developed by Wang et al., which simultaneously captures carbonyl‐, thiol‐, and amine‐containing molecules for quantification down to subnanogram per milliliter concentrations.^58^ An alternative post‐column derivatization scheme has been recently reported for detecting difficult‐to‐ionize metabolites containing hydroxyl groups. This method uses 2‐(4‐boronobenzyl) isoquinolin‐2‐ium bromide as a binding reagent and increases sensitivity up to 43‐fold compared to underivatized counterparts.^59^

While chemoselective functionalization can greatly improve the limits of detection, there are several inherent limitations with all probe‐based approaches. First, they necessitate the use of specialized probes with multistep synthesis and require a reactive functional group to be captured or detected. Moreover, even if a molecule contains a taggable group, it may not be effectively derivatized or extracted under the conditions used. Therefore, chemoselective metabolite enrichment is envisioned to be complementary and performed in addition to traditional LC‐MS–based metabolomics so that as much information as possible can be captured from a single experiment. Finally, chemoselective derivatization cannot help determine the biosynthetic origin of the molecule, outside of highlighting certain functional groups that may be classically host‐ or microbially derived. The examples discussed above are not specific to metabolites produced by the gut microbiota. Therefore, complementary strategies are needed to help identify the biosynthetic and ecological origins of important metabolites in the gut.

### Genome‐guided synthesis

Genome‐guided strategies were developed to identify potential biosynthetic producers and reduce ambiguity about where a metabolite can come from. In these workflows, biosynthetic gene clusters are identified using various genomic analysis workflows then chemical structures are predicted, synthesized, and validated using comparative bioactivity assays, NMR, MS, and/or heterologous expression (Figure 2A, middle).

A workflow combining bioinformatics with chemical synthesis was developed by Chu et al. to predict and discover new peptides called humimycins from the gut microbiome. In this approach, synthetic bioinformatic natural products (syn‐BNPs) are predicted based on nonribosomal peptide synthase (NRPS) gene clusters in human‐associated gut bacteria, synthesized, and then tested for antibiotic activity.^60^, ^61^ The authors initially targeted 30 predicted linear peptides using Fmoc‐based solid phase peptide synthesis and obtained 25 in pure form that were tested against human commensal and pathogenic bacteria. The humimycins were discovered using this workflow and found to be broadly active against *Firmicutes* with higher efficacy against *Staphylococcus* and *Streptococcus* genera. Furthermore, humimycin A **(8)** and B were shown to modulate beta‐lactam activity, transforming previously inactive antibiotics into potent antimicrobial agents and offering a promising step forward in antibiotic drug therapy. Treating an methicillin‐resistant *Staphylococcus aureus* (MRSA) infection with combined dicloxacillin and humimycin significantly increased the survival of peritonitis‐sepsis model mice compared to administering either drug alone. This syn‐BNP strategy was later expanded to predict cyclic peptides from NRPS gene clusters. In this study, 157 cyclic peptides were synthesized based on predictions from 96 unique gene clusters, then they were screened for antibiotic activity, nine of which showed narrow or broad‐spectrum antimicrobial activity against antibiotic‐resistant pathogens. These syn‐BNP examples demonstrate how the integration of genomics analysis with high‐throughput synthesis and screening technologies can enable the discovery of novel therapeutic agents from the gut microbiome.

An alternative genomic‐centered approach was implemented by Schneider et al. for the discovery of ruminopeptin **(9)** from *Ruminococcus bromii*, in which human gut metagenomic data was mined for NRPS biosynthetic gene clusters.^62^ In vitro characterization of *rup‐*encoded biosynthetic enzymes was then performed in combination with synthetic chemistry and bioactivity assays to elucidate the structure. Ultimately, the *R. bromii‐*derived metabolites were identified as dipeptide aldehydes. Chemical synthesis was essential in this study because exhaustive attempts to isolate the predicted products had already been made using culturing and in vitro biochemistry with no success. Just prior to publication, Fischbach and coworkers reported the presence of dipeptide aldehydes in the gut microbiome that were isolated as the pyrazinone byproducts due to nonenzymatic cyclization and oxidation reactions.^63^ Evidence from Schneider et al. supports that aldehydes are the active form of these gut microbial metabolites. The discovery of ruminopeptin highlights the challenges associated with isolation‐dependent methods and demonstrates how chemical synthesis can be used to overcome these limitations.

The story of colibactin **(10)** structure elucidation is perhaps the most noteworthy example of how synthesis and genomics can be combined, alongside other tools to identify even the most elusive structures. Colibactins are hybrid polyketide‐nonribosomal peptide enterotoxins, which are associated with colorectal cancer and produced by *E. coli* possessing *clb* genes. Due to their molecular instability, the structure of colibactin remained an enigma until recently and was deduced only through an integrated and iterative approach combining synthesis with genomic and metabolomic technologies. The DNA‐damaging effects of colibactin‐producing (*clb* ^+^) *E. coli* were first discovered in 2006, but the mechanism by which genotoxicity occurred was unknown at the time.^64^ In 2018, the mechanism by which the clb gene cluster damaged DNA was determined to be through interstrand cross‐linkages (ISCs), but the chemical structure of the colibactin toxin still had not been established.^65^ Li et al. then uncovered the precise molecular mechanism behind colibactin‐induced ISCs, finding that genotoxic colibactin metabolites induce a DNA double‐strand break through a copper‐mediated oxidative cleavage mechanism.^66^ Enzymology, bioinformatics, stable isotope feeding, biosynthesis experiments, and genomic editing studies provided many insights into colibactin's bioactivity and biosynthesis, including that there were two DNA‐reactive sites, but the structure remained unknown.

In 2019, Herzon and coworkers completed the puzzle and provided the absolute structure of colibactin using a clever combination of genetics, organic synthesis, isotope labeling, metabolomics, and chemical probe‐mediated capture.^67^ Since colibactin had previously eluded isolation, their studies focused on identifying the DNA‐linked adducts using MS. Wild‐type *clb^+^ E. coli* was cultured in media supplemented with isotopically labeled amino acids, glucose, and/or ammonium chloride to deduce colibactin's biosynthetic building blocks. Assigned stereochemistry was based on known intermediates in addition to nonribosomal peptide synthase and polyketide synthase biosynthetic logic. In parallel, Balskus and coworkers implemented a complementary synthesis‐independent approach which confirmed the assigned structure.^68^

These initial studies enabled further development of synthesis‐mediated detection and therapeutic strategies for colibactin‐associated colon cancer. To aid in the clinical detection of colibactin‐producing *E. coli* strains, Watanabe et al. developed a simple, high‐throughput readout using loop‐mediated isothermal amplification technology and a synthetic ClbP‐activated fluorescent probe. The authors isolated wild‐type, high‐colibactin–producing *E. coli* strains from colorectal cancer patients using this method.^69^ Moreover, Balskus and coworkers  recently designed boronic acid‐based synthetic inhibitors targeting colibactin biosynthesis, which mimic the biosynthetic precursor precolibactin and effectively inhibit the colibactin‐activating peptidase ClbP.^70^

Genomic data can also be integrated with metabolic modeling to identify molecular interactions between microbes and their hosts. Genome‐scale metabolic models (GEMs) were initially developed to investigate metabolic flux in individual organisms but can now be used to compute complex metabolic networks for entire microbial communities.^71^, ^72^ Constraint‐based reconstruction and analysis modeling of bacterial consortia combines individual GEMs to simulate the metabolism of microbial communities, and can even be used to explore how metabolic flux and growth behavior respond to differing nutrient inputs.^73^, ^74^, ^75^, ^76^ Tools like BioTransformer 3.0 incorporate host biotransformations into metabolic models, providing a more comprehensive analysis of gut metabolism.^77^ Semi‐automated workflows such as CarveMe, MetaGEM, MIGRENE, and gapseq were developed to assist in metabolic reconstruction, generating community metabolic models from whole genome sequences or short‐read metagenomics data.^78^, ^79^, ^80^, ^81^ Moreover, AGORA2 is a publicly available collection of genome‐scale reconstructions that were extracted from whole‐genome sequencing data for thousands of cultured microbial strains and can be used to predict personalized metabolism.^82^ Heinken et al. expanded this resource to include data from uncultured microbes in a new collection called APOLLO, which contains freely available sample‐specific microbiome community models generated from a semi‐automated reconstruction of 247,092 metagenomic assembled genomes.^83^ While these methods are not synthesis‐focused, community metabolic models are integral to unraveling how microbial communities allocate and metabolize resources, and also provide a high‐throughput means for identifying metabolite–microbe associations.

### Reverse metabolomics

Most recently, a strategy called reverse metabolomics was created which combines organic synthesis with untargeted metabolomics data mining to uncover new host and microbial metabolites.^46^ In this approach, potential metabolites of interest are synthesized and analyzed using MS/MS, and then their MS/MS spectra are searched for in public metabolomics data (Figure 2A, bottom). This method was developed by Gentry et al. to identify whether metabolites are found in gut or fecal samples and how frequently they are detected in healthy versus diseased individuals. Reverse metabolomics was first implemented to analyze a variety of conjugated bile acids, *N*‐acyl amides, and fatty acid conjugated hydroxy fatty acids and also to provide insight into where these compounds are found in public LC‐MS/MS data in terms of sample types, species, and phenotype.^46^, ^84^, ^85^ Thousands of unique structures were synthesized in this study, then searched for in the Global Natural Products Social Molecular Networking (GNPS) database using Mass Spectrometry Search Tool (MASST) and ReDU.^84^, ^86^, ^87^ Reverse metabolomics enables accessible and high‐throughput searching of biochemical space to find novel disease‐metabolite associations relevant to human health.^46^, ^88^ Using reverse metabolomics, Gentry et al. determined that many of the synthesized bile amidates were detected more frequently in individuals with inflammatory bowel disease (IBD) than in healthy controls, particularly for those structures containing trihydroxylated bile acid cores. This finding was significant as it was consistent across several cohorts and suggests that certain bile amidates are elevated in IBD. This hypothesis was validated using multiple independent clinical IBD cohorts, where most cholic and chenodeoxycholic acid amidates were found to be significantly increased in humans with IBD versus healthy controls. Targeted studies determined that bile amidates were elevated in individuals with active Crohn's disease, highlighting a possible connection to disease‐related pathways. Therefore, the biological activities of these metabolites were further examined and some bile amidates like Phe‐conjugated chenodeoxycholic acid **(11)** were found to be immunomodulating compounds and ligands for host receptors expressed in the gut.^46^, ^89^

The class of bile amidate metabolites reported from this study originates from the gut microbiota, where microbes with the bile salt hydrolase (BSH) gene form these MCBAs.^89^, ^90^ Since the initial discovery of MCBAs, many others have reported on the identification, production, and bioactivity of these compounds now that they are part of known metabolic pathways.^91^, ^92^, ^93^ Reverse metabolomics has a high‐throughput capacity to unlock new biochemical spaces and metabolic transformations. For example, the MCBAs discovered using reverse metabolomics are close analogs to known bile amidates and products of a well‐known enzyme, BSH; nonetheless, these structures eluded detection for well over a century. Therefore, we envision that reverse metabolomics can uncover other simple but “unusual” transformations of bioactive compound classes widespread in the human gut. Through this chemistry‐based interrogation of biological systems, annotation rates in untargeted metabolomics can greatly improve and our knowledge of secondary metabolism will be more complete. Furthermore, by leveraging repository‐scale meta‐analysis of public metabolomics data, reverse metabolomics enables researchers to uncover robust metabolite–phenotype associations that are detected across multiple cohorts. In the future, reverse metabolomics may be combined with genome mining and community modeling tools for most effective exploration of gut microbial metabolism.

## CONCLUSION

Synthetic chemists provide absolute stereochemical structure for novel natural products and supply material for biological assays. Conversely, natural product chemists and biological researchers continuously inspire synthetic chemists with new proposed structures and provide “mountains to summit,” where challenging architectures require creative and perhaps risky maneuvers.^94^, ^95^ Natural product studies greatly benefit from synthesis and vice versa, and this synergism feeds pharmaceutical development and drug design.^96^ The gut microbial metabolome is a new frontier for drug discovery with many new targets, biomarkers, and molecular scaffolds yet to be found. The future of medicine has bright potential, where any health condition may be diagnosed and treatment monitored from a standard lab test. Imagine a reality where we can detect increasing levels of disease‐causing metabolites in real time and perhaps even intercept their production. Metabolomics could be at the heart of this personalized health care vision where comprehensive analysis of small molecules in blood, urine, or fecal samples is performed in standard clinical settings. However, this dream will not be possible without available extensive libraries of chemical standards and reference spectra. This review presents examples where synthesis was used to extend known biochemical space in hopes of inspiring new methods, collaborations, and applications for this purpose.

## AUTHOR CONTRIBUTIONS

A.J.K. and N.K.N. contributed equally to this work. All authors contributed to the writing and editing of the manuscript.

## COMPETING INTERESTS

The authors declare no competing financial interests.

## Data Availability

Data sharing is not applicable to this article as no datasets were generated or analyzed during the current study.
